# Albumin–globulin ratio is a predictive biomarker of antitumour effect of immune checkpoint inhibitors in cancer patients

**DOI:** 10.1080/07853890.2025.2591219

**Published:** 2025-11-24

**Authors:** Kaixiang He, Fujiang Xu, Li Xiang, Yuhao Luo

**Affiliations:** Department of Oncology, The Affiliated Hospital of Southwest Medical University, Sichuan, Luzhou, China

**Keywords:** Immune checkpoint inhibitors, renal cell carcinoma, cancer, prognosis, therapeutic response

## Abstract

**Background:**

Immune checkpoint inhibitors (ICIs) have transformed cancer therapy, yet the heterogeneity of treatment responses underscores the need for reliable prognostic biomarkers. The albumin-to-globulin ratio (AGR), an indicator of systemic inflammation and nutritional status, has emerged as a potential predictor of ICI outcomes. This study aimed to systematically evaluate the prognostic significance of AGR in patients receiving ICIs through a meta-analysis and to validate the findings in a single-centre cohort.

**Methods:**

A systematic literature search was conducted using PubMed, EMBASE, and the Cochrane Library to identify studies published prior to June 6, 2025. The primary endpoints were overall survival (OS), progression-free survival (PFS), and disease control rate (DCR). In addition, a retrospective analysis was performed on a cohort of 74 patients with renal cell carcinoma (RCC) treated with ICIs at our institution to assess the prognostic value of baseline AGR in relation to OS and PFS.

**Results:**

Seven studies encompassing 1,460 patients were included in the meta-analysis. Higher pretreatment AGR was significantly associated with improved OS (HR = 0.44; 95% CI: 0.30–0.66; *p* < 0.001), extended PFS (HR = 0.61; 95% CI: 0.53–0.71; *p* < 0.001), and superior DCR (OR = 4.48; 95% CI: 2.58–7.77; *p* < 0.001). Sensitivity analyses confirmed the robustness of these associations. In our institutional RCC cohort, elevated AGR was independently linked to prolonged OS (*p* = 0.017) and PFS (*p* = 0.030), consistent with findings from the pooled data.

**Conclusion:**

AGR is a simple, inexpensive, and non-invasive biomarker with significant prognostic value in patients undergoing ICI therapy. These findings support its potential role in guiding clinical decision-making and optimizing patient selection for immunotherapy.

## 1. Introduction

Immune checkpoints serve as crucial modulators of immune homeostasis by transmitting either activating or suppressive cues, thereby contributing to immune tolerance and enabling malignant cells to evade immunosurveillance [1–3]. Over the past decade, cancer immunotherapy has witnessed transformative advances, most notably through the development and clinical application of immune checkpoint blockade therapies [4–6]-. Unlike traditional cytotoxic regimens—such as chemotherapy and radiation—that directly target rapidly dividing tumour cells, immune checkpoint inhibitors (ICIs) represent monoclonal antibodies designed to antagonize inhibitory pathways involving PD-1, PD-L1, or CTLA-4 [4,5]. By disrupting these immune-inhibitory interactions within the tumour microenvironment, ICIs alleviate T-cell exhaustion and reinvigorate effector responses. This immunologic reprogramming restores the cytotoxic potential of tumor-infiltrating lymphocytes, thereby enhancing endogenous antitumor immunity and improving clinical outcomes across multiple cancer types [4,5].

Response rates to ICI therapy exhibit substantial variability across malignancies, with clinical benefit observed in only approximately 10% to 40% of cases depending on tumour type. Notably, even among patients who initially respond favourably, disease progression commonly ensues over time [7,8]. Moreover, the immune-related adverse events induced by ICI treatment can range from manageable toxicities to severe, and occasionally fatal, complications [9]. These limitations underscore the imperative for early stratification strategies capable of identifying individuals unlikely to benefit from immunotherapy, thereby minimizing unnecessary exposure to ineffective regimens and mitigating potential harms [10,11].

Currently, PD-L1 expression assessed *via* immunohistochemistry in tumour biopsies is the most widely utilized biomarker to guide ICI-based decision-making [12]. However, the utility of PD-L1 as a predictive tool remains suboptimal in clinical settings due to its spatial and temporal heterogeneity across tumour microenvironments. Alternative biomarkers—such as tumour mutation burden and microsatellite instability—have been proposed as companion diagnostics for specific agents, yet their predictive accuracy when applied individually remains modest [13,14]. Compounding this challenge, the lack of consensus on standardized thresholds and assay methodologies further complicates the reproducibility and comparability of biomarker-based evaluations. Therefore, the identification and validation of novel, reliable prognostic indicators are urgently needed to refine patient selection for ICI therapy and optimize clinical outcomes in oncology.

In the light of the recognized challenges and intrinsic limitations of tissue-derived biomarkers in the context of ICI therapy, attention has increasingly shifted towards circulating markers—particularly those derived from routine hematologic and biochemical profiles that mirror systemic immune-inflammatory dynamics and possess prognostic relevance [15]. Accumulating evidence has linked elevated levels of C-reactive protein (CRP), albumin, as well as disproportionate neutrophil-to-lymphocyte (NLR) and platelet-to-lymphocyte ratios (PLR), to unfavourable clinical outcomes, reflecting heightened inflammatory burden in patients undergoing ICI treatment [10,16–18]. Likewise, the albumin-to-globulin ratio (AGR), while originally investigated as a surrogate for systemic inflammation, also offers the added advantage of indicating nutritional reserves, though its clinical utility remains underexplored in ICI-treated populations [19]. Based on these insights, this study attempts to systematically examine the relationship between AGR levels and survival indicators of patients receiving ICIs using meta-analysis and cohort data from our centre.

## 2. Methods

### 2.1. Search strategy, inclusion criteria, and exclusion criteria for the meta-analysis

This meta-analysis was meticulously designed and executed in alignment with the PRISMA (Preferred Reporting Items for Systematic Reviews and Meta-Analyses) framework [20]. To ensure a comprehensive capture of relevant literature, we implemented a systematic search strategy across three principal biomedical databases—PubMed, EMBASE, and the Cochrane Library—encompassing publications available up to June 6, 2025. To identify relevant literature with maximal sensitivity and specificity, a set of carefully curated search terms was employed. These included Medical Subject Headings (MeSH) and keyword variants related to immune checkpoint blockade and tumour markers. Terms incorporated into the search strategy encompassed: ‘immune checkpoint inhibitors’ [MeSH], as well as commonly used nomenclature such as ‘ICIs’, ‘PD-1 inhibitors’, ‘PD-L1 inhibitors’, and ‘CTLA-4 inhibitors’. Additionally, specific agents were queried individually, including pembrolizumab, nivolumab, ipilimumab, atezolizumab, camrelizumab, sintilimab, tislelizumab, toripalimab, and envafolimab. To capture studies examining tumour biomarker correlations, ‘albumin to globulin ratio’ were also integrated into the search. A full account of the search methodology is provided in Supplementary material 1. To augment retrieval sensitivity, reference lists of all eligible articles were further screened manually for additional pertinent studies that may not have been indexed through database queries. Study selection was conducted independently by two investigators, thereby reducing the likelihood of selection bias and enhancing the objectivity of inclusion. Ethical approval was not required for this study because it involved the retrieval and synthesis of data from previously published studies.

Eligibility for inclusion was determined according to the following pre-specified criteria: (1) observational studies, whether retrospective or prospective in design, evaluating the prognostic significance of the AGR in relation to clinical endpoints such as overall survival (OS), progression-free survival (PFS), or disease control rate (DCR); (2) stratification of patient cohorts based on AGR thresholds into high and low groups; (3) provision of effect estimates—hazard ratios (HRs) and/or odds ratios (ORs)—accompanied by 95% confidence intervals (CIs); and (4) availability of the full article in English. Studies were excluded if they fell into any of the following categories: (1) duplicate reports or overlapping datasets presented in multiple publications; or (2) non-original research formats such as case reports, conference proceedings, narrative reviews, editorials, expert commentaries, or clinical practice guidelines. Also, Non-English languages and studies with secondary analysis were excluded. When redundant datasets were identified, preference was given to the study offering the most granular data and demonstrating methodological rigor, as assessed by internal quality criteria [21].

### 2.2. Data extraction and quality assessment for the meta-analysis

During the data abstraction process, we systematically collected key methodological and clinical attributes from each eligible publication. This included the name of the lead investigator, year of publication, study timeline, geographic location, cancer subtype under investigation, treatment approach, sample size, baseline demographics (such as patient age and sex), and the predefined cutoff values used to stratify AGR. When available, hazard ratios (HRs) derived from multivariate regression analyses were preferentially extracted; in the absence of such data, estimates obtained from univariate models or reconstructed from Kaplan–Meier curves were incorporated instead. To assess the methodological rigor of non-randomized studies, the Newcastle–Ottawa Scale (NOS) was applied. Studies achieving a NOS score of six or higher were considered to exhibit satisfactory methodological robustness.

### 2.3. Study cohort and data collection for the retrospective study

A retrospective cohort investigation was undertaken utilizing patient data archived at our medical centre, with the primary aim of assessing the prognostic implications of pretreatment AGR in individuals diagnosed with renal cell carcinoma (RCC). Institutional ethics committee approval was secured in advance, and given the observational and non-interventional design of the study, the requirement for informed consent was formally waived.

The study population comprised RCC patients who received ICIs—either targeting the PD-1 or PD-L1 axis—between 2011 and 2023. Inclusion criteria stipulated the availability of at least one quantifiable tumour lesion, as defined by the Response Evaluation Criteria in Solid Tumours (RECIST) version 1.1. Participants were excluded from analysis if they had previously been exposed to ICIs or if baseline AGR values were unavailable at treatment initiation.

### 2.4. Statistical methods

Categorical data were summarized as absolute frequencies accompanied by their respective proportions. To evaluate survival distributions across comparison groups, Kaplan–Meier survival estimators were generated, and intergroup differences were analysed using the Cox proportional hazards regression framework.

Meta-analytical procedures were executed using Stata version 18.0. Forest plots were constructed to visually depict effect size estimates and confidence intervals. Between-study heterogeneity was rigorously evaluated through both Cochran’s Q statistic and the I^2^ metric, with substantial inconsistency defined by an I^2^ exceeding 50% or a Q-test *p*-value below 0.1 [22]. In scenarios where notable heterogeneity was observed, the DerSimonian–Laird random-effects model was utilized to accommodate inter-study variability. Alternatively, when heterogeneity was minimal, pooled estimates were calculated *via* the inverse-variance weighted fixed-effects model.

To interrogate the potential presence of publication bias, Begg’s funnel plot and Egger’s regression asymmetry test were both applied [23]. The stability of the meta-analytic results was interrogated through leave-one-out sensitivity analysis, whereby the exclusion of individual studies was iteratively tested to gauge their influence on the overall effect size [24]^.^ A two-tailed *p*-value less than 0.05 was considered indicative of statistical significance.

## 3. Results

### 3.1. Search results and study characteristics

**Figure 1.**
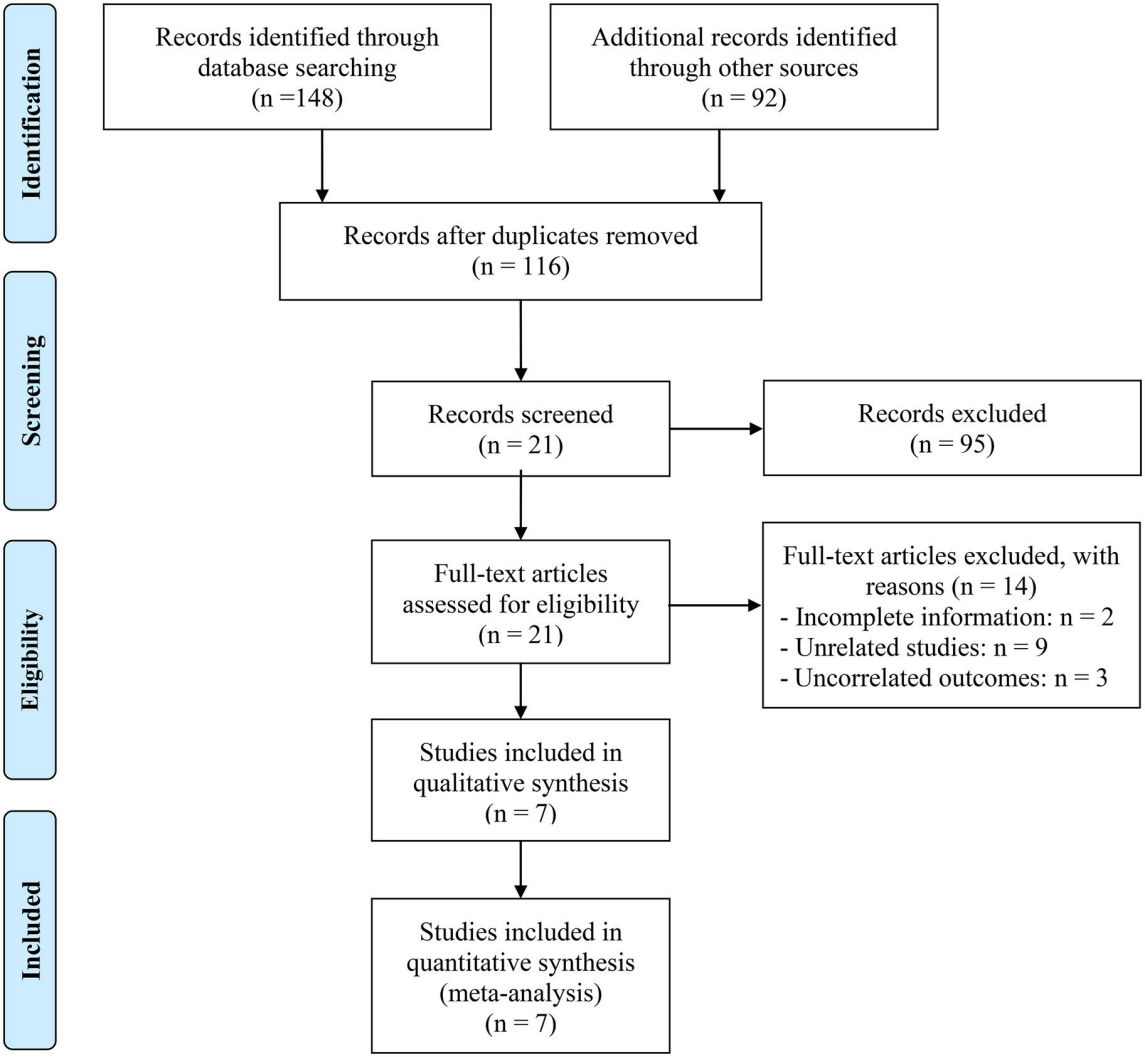
The flow diagram of identifying eligible studies.

Through an extensive initial database query, augmented by manual evaluation of bibliographic references, a total of 240 candidate publications were retrieved. After excluding 124 repeated entries, 95 records were eliminated based on title and abstract screening due to inconsistency with the predefined eligibility parameters. Subsequently, 21 articles underwent full-text review; of these, 14 failed to satisfy the requisite inclusion thresholds. Ultimately, a total of seven studies met all methodological criteria and were incorporated into the final quantitative synthesis [19,25–30] (Figure 1).

Table 1 provides a comprehensive overview of the core characteristics of the studies incorporated into the present meta-analysis. Collectively, these investigations encompassed 1,460 patients, with sample sizes per study ranging from 74 to 544 individuals. Among the selected publications, three addressed diverse tumor types (pan-cancer), whereas two were specifically dedicated to non-small cell lung cancer (NSCLC). All five studies employed retrospective designs. Based on assessment using the Newcastle–Ottawa Scale (NOS), the methodological quality of the included research was deemed moderate to high, with scores spanning from 6 to 7—indicating a relatively low risk of systematic bias (Table 1).

**Table 1.** Main characteristics of the studies included.

Study	Study period	Cancer type	Country	Sample size	Gender (male/female)	Age	Treatment	Cut-off	NOS
Zhou et al. 2025	10/2010-10/2021	NSCLC	China	544	428/116	287/257	ICI	1.21	7
Wang et al. 2023	01/2020-10/2021	Gastric cancer	China	195	135/60	61.4 (29–84)^b^	PD-1 inhibitors	1.40	6
Zhang et al. 2022	10/2018-05/2021	Pan-cancer	China	149	75/67	64 (23–85)^b^	Anti-PD-1/PD-L1 antibodies	1.24	6
Ma et al. 2022	01/2017-05/2020,	Pan-cancer	China	95	66/29	62 (30–80)^a^	Anti-PD-1/PD-L1	0.90	7
Guven et al. 2022	01/2014-08/2020	Pan-cancer	Turkey	253	145/67	61 (51–67)^a^	ICIs	1.21	7
Taguchi et al. 2021	01/2018-07/2020	Urothelial carcinoma	Japan	150	111/39	71 (66–76)^a^	Pembrolizumab	0.95	6
Nakanishi et al. 2020	09/2015-04/2018	NSCLC	Japan	74	52/22	66.5 (39–85)^a^	Nivolumab or pembrolizumab	1.17	6

^a^ median (IQR), ^b^median (range), ^c^age > 60 vs. ≤ 60. ICIs, immune checkpoint inhibitors; NSCLC, non small cell lung cancer; PD-1, programmed cell death protein-1; PD-L1, programmed cell death ligand-1.

### 3.2. Baseline albumin-to-globulin ratio and overall survival

**Figure 2.**
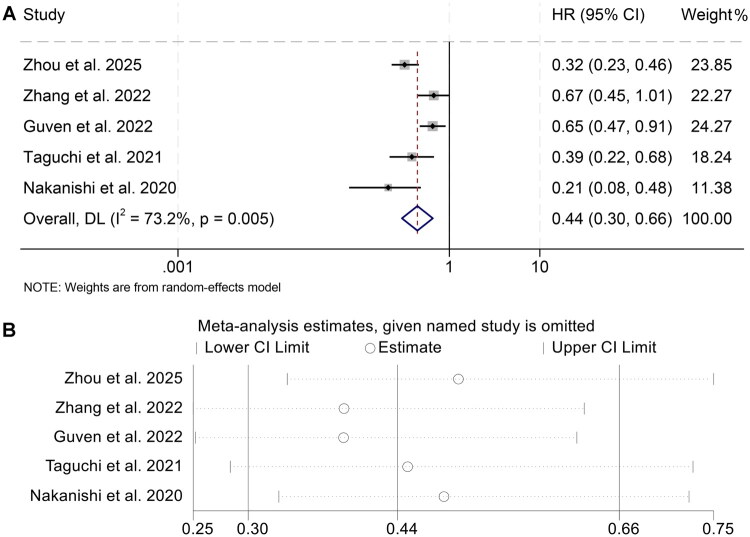
Figure A displays forest plots summarizing the relationship between pretreatment albumin-to-globulin ratio and overall survival among individuals receiving immune checkpoint blockade. Figure B illustrates the sensitivity analysis conducted to evaluate the consistency and robustness of this association across included studies. Abbreviations: HR, hazard ratio; CI, confidence interval.

A total of five studies (1170 patients) met the eligibility criteria and were incorporated into this meta-analysis to rigorously assess the prognostic relevance of the AGR for OS in cancer patients undergoing ICIs. The synthesized HR demonstrated a significant survival advantage associated with higher AGR levels (HR = 0.44; 95% CI: 0.30–0.66; *p* < 0.001; Figure 2(A)).

Assessment of heterogeneity across studies revealed substantial inter-study variability, as evidenced by an I^2^ value of 73.2% and a statistically significant Cochran’s Q test (*p* = 0.005), thereby warranting the application of a random-effects meta-analytic model.

To verify the stability of the effect estimate, leave-one-out sensitivity analysis was conducted, which consistently reproduced the overall trend, indicating minimal influence from individual studies (Figure 2(B)). Furthermore, publication bias was evaluated through Begg’s and Egger’s tests, neither of which indicated significant asymmetry (*p* = 0.462 and *p* = 0.435, respectively), suggesting a low risk of reporting bias.

Subgroup analyses showed that both univariate and multivariate models consistently demonstrated a strong association between higher AGR values and longer OS outcomes (Figure S1A).

### 3.3. Baseline albumin-to-globulin ratio and progression-free survival

**Figure 3.**
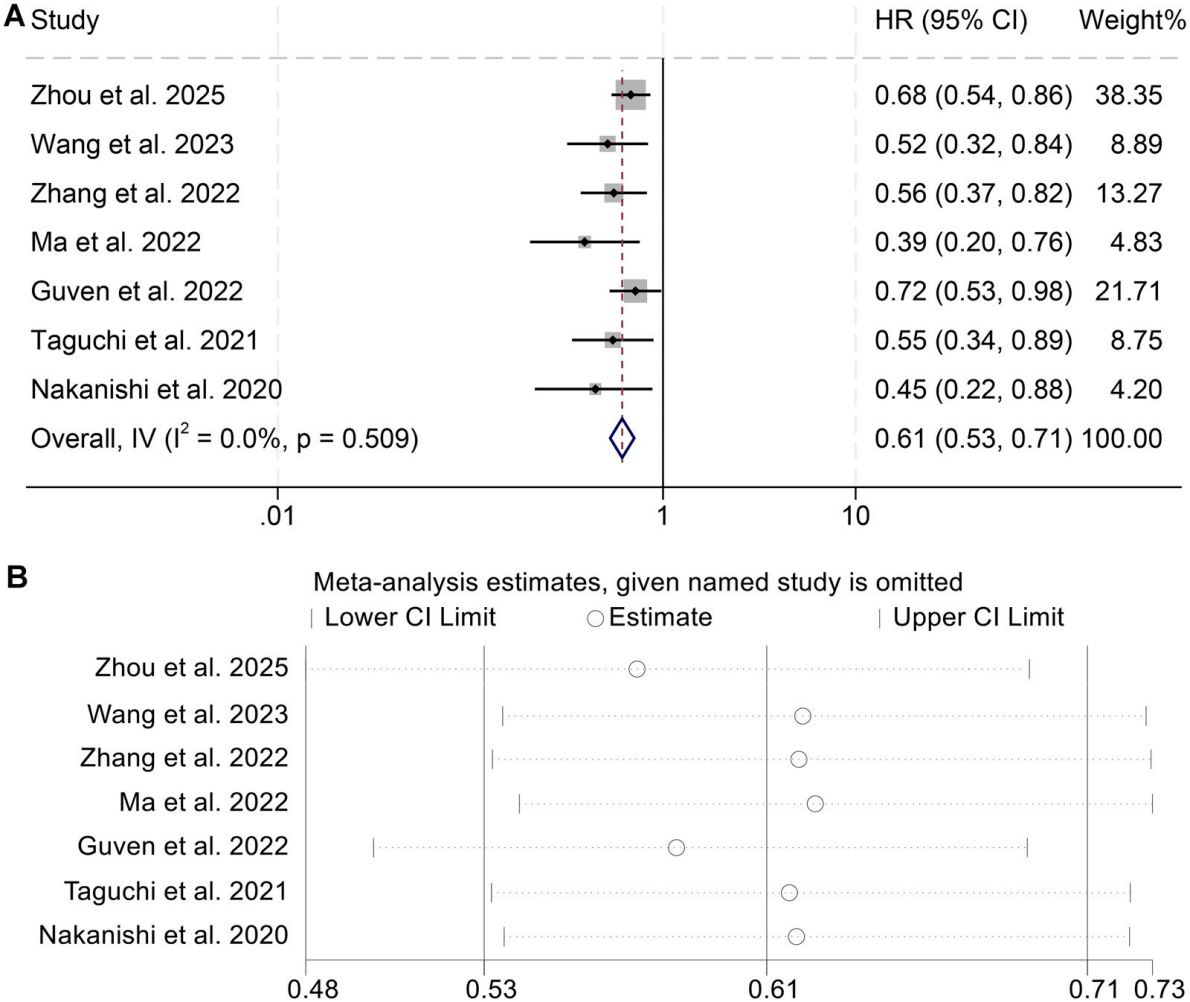
Figure A displays forest plots summarizing the relationship between pretreatment albumin-to-globulin ratio and progression-free survival among individuals receiving immune checkpoint blockade. Figure B illustrates the sensitivity analysis conducted to evaluate the consistency and robustness of this association across included studies. Abbreviations: HR, hazard ratio; CI, confidence interval.

A total of 1,460 cancer patients across seven independent cohorts were collectively assessed to elucidate the prognostic relevance of the AGR in relation to PFS. Meta-analytical synthesis demonstrated that individuals with elevated AGR values experienced significantly prolonged PFS outcomes (HR = 0.61, 95% CI: 0.53–0.71, *p* < 0.001; Figure 3(A)). Given the absence of appreciable heterogeneity among included studies (I^2^ = 0, *p* = 0.509), a fixed-effect modelling framework was applied to derive pooled estimates.

To examine the stability of this association, a leave-one-out sensitivity analysis was performed. The exclusion of individual studies did not materially alter the overall HR, thereby supporting the internal consistency of the findings (Figure 3(B)). Furthermore, assessments of publication bias using both Begg’s and Egger’s statistical approaches yielded no significant evidence of asymmetry (Begg’s test *p* = 0.172; Egger’s test *p* = 0.103), minimizing concern for selective reporting across the included literature.

Subgroup analyses showed that both univariate and multivariate models consistently demonstrated a strong association between higher AGR values and longer PFS outcomes (Figure S1B).

### 3.4. Pre-immunotherapy albumin-to-globulin ratio and disease control rate

**Figure 4.**
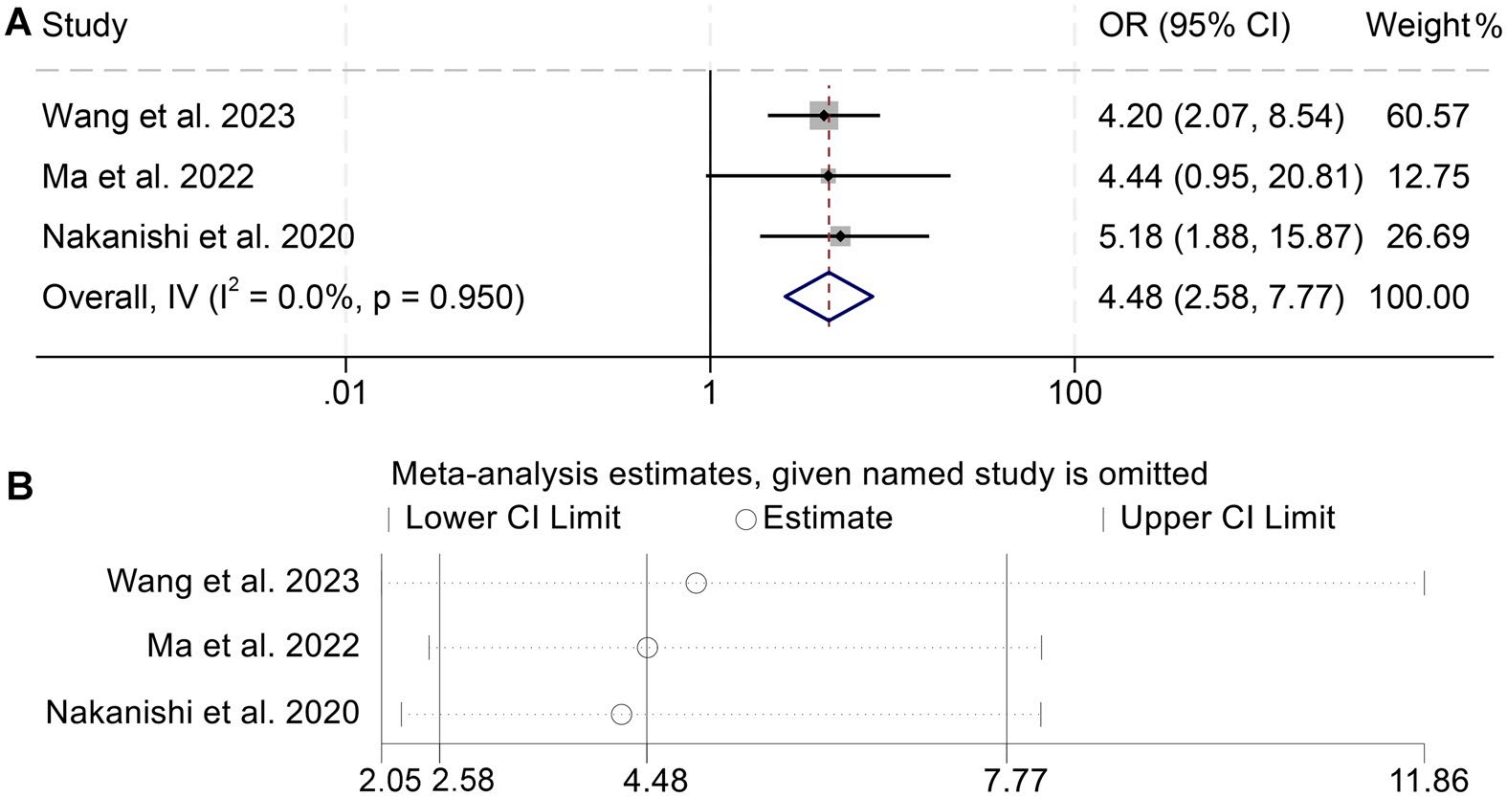
Figure A presents a meta-analytic forest plot delineating the association between baseline albumin-to-globulin ratio and disease control rate in patients undergoing treatment with immune checkpoint inhibitors. Figure B depicts the results of sensitivity analyses performed to verify the reproducibility and resilience of this observed correlation across the included studies. Abbreviations: OR, odd ratio; CI, confidence interval.

The relationship between the AGR and DCR was quantitatively assessed based on data derived from three independent cohorts comprising a total of 364 individuals diagnosed with malignancies. Given the absence of notable heterogeneity among the selected studies (I^2^ = 0, *p* = 0.950), a fixed-effects statistical framework was deemed appropriate for data synthesis. Meta-analytic integration revealed that patients exhibiting elevated AGR values experienced significantly enhanced treatment responses, as reflected by a markedly improved DCR (OR = 4.48, 95% CI: 2.58–7.77, *p* < 0.001; Figure 4(A)), compared to counterparts with lower baseline AGR. To examine the reliability of this association, leave-one-out sensitivity analyses were performed, which yielded consistent effect sizes across iterations, thereby reinforcing the stability and credibility of the observed findings (Figure 4(B)).

### 3.5. Baseline albumin-to-globulin ratio and prognosis in our cohort

In the light of the limited evidence assessing the prognostic relevance of the AGR in patients with RCC, we conducted a retrospective investigation within our institution to advance the evolving understanding of AGR as a predictive biomarker in RCC cohorts.

Supplementary Table 1 outlines the baseline demographic and clinical features of the 74 RCC patients included in this internal investigation. The median age at diagnosis was 56.4 years (range: 34.7–79.6 years), with male patients comprising 67.57% (*n* = 50) of the study population. Regarding performance status, as determined by the Eastern Cooperative Oncology Group (ECOG) scale, 54.06% (*n* = 40) of patients were classified as ECOG 0, 33.87% (*n* = 25) as ECOG 1, and 12.16% (*n* = 9) as ECOG 2. Based on stratification using the International Metastatic RCC Database Consortium (IMDC) criteria, 51.35% (*n* = 38) were categorized into risk group 0, 36.49% (*n* = 27) into group 1, and the remaining 12.16% (*n* = 9) into group 2.

**Figure 5.**
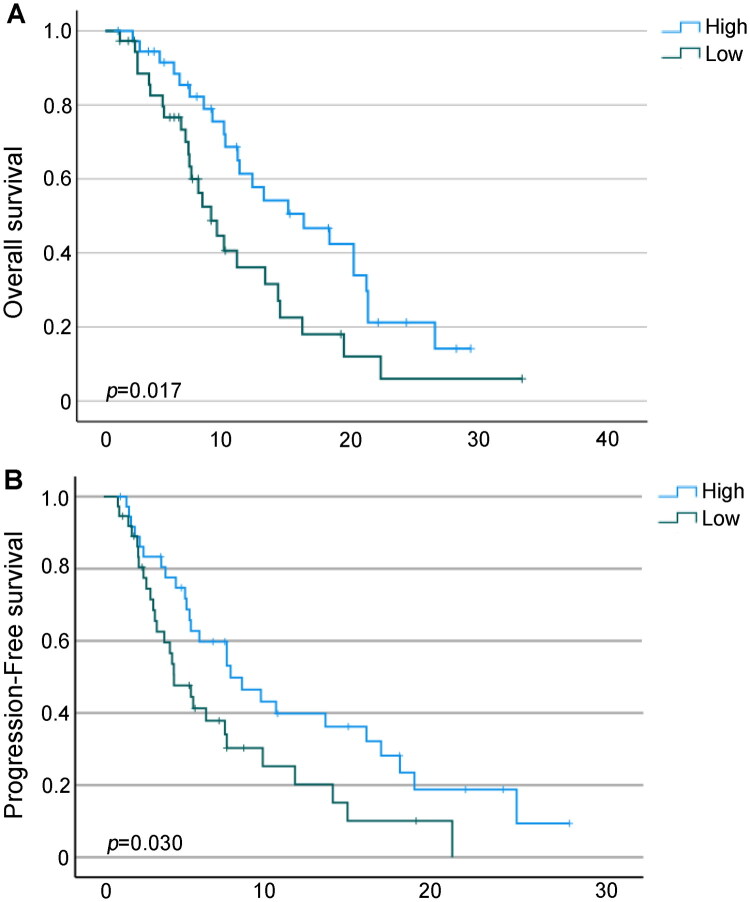
Kaplan–Meier survival estimates for overall survival (A) and progression-free survival (B) are presented, stratified by albumin-to-globulin ratio levels in our cohorts.

Patients were dichotomized into high and low AGR subgroups based on the median pretreatment AGR value (median follow-up: 24.6 months; range: 1.4–130.9 months). Kaplan–Meier analysis demonstrated that patients with elevated baseline AGR had significantly longer OS (*p* = 0.017; Figure 5(A)) and PFS (*p* = 0.030; Figure 5(B)) compared with those in the low AGR group.

## 4. Discussion

As a biomarker obtainable from routine serum assays, the AGR offers a pragmatic and economically feasible alternative for clinical use. In the present analysis, elevated AGR values were robustly associated with improved prognostic outcomes among individuals with malignancies. These findings were further corroborated by evidence from our institutional cohort, reinforcing the prognostic relevance of AGR within this therapeutic context.

To our knowledge, this meta-analysis represents the first comprehensive evaluation identifying the AGR as a prognostic biomarker for the therapeutic efficacy of anti-PD-1 antibodies in individuals with NSCLC [31]. Albumin and globulin—key constituents of serum proteins—collectively form the basis of AGR. The globulin fraction, which forms the denominator of this ratio, comprises numerous immune-associated molecules including immunoglobulins, CRP, interleukins, tumour necrosis factor, and transforming growth factor-β. These inflammatory mediators are frequently upregulated in patients with malignancy-driven chronic inflammation [32,33]. Evidence suggests that such cytokines facilitate tumour growth and mediate resistance to chemotherapy by promoting both neoplastic proliferation and angiogenesis [32,34].

Based on this biological rationale, we postulated that diminished globulin levels—manifested as a higher AGR—may indicate reduced systemic inflammation, thereby correlating with a more favourable response to anti-PD-1 therapy [35]. Conversely, albumin, forming the numerator of AGR, is a multifunctional hepatic protein integral to homeostasis and widely acknowledged as a surrogate for nutritional and inflammatory status [31,32]. Hypoalbuminemia, often reflective of systemic malnutrition, has been linked to compromised immune surveillance, weakened phagocytosis, and impaired cell-mediated immunity in cancer patients. Additionally, inflammation or neoplastic burden itself can lead to reduced albumin synthesis, making hypoalbuminemia a recognized predictor of adverse clinical outcomes in oncology [31,32].

Thus, patients with elevated serum albumin—that is, a high AGR—may retain a more robust immunological and nutritional profile, positioning them to derive greater clinical benefit from PD-1 blockade. Nevertheless, it is important to acknowledge that albumin levels may be influenced by multiple non-neoplastic factors such as physiological stress, hepatic dysfunction, and aging, potentially confounding its independent predictive value. In the light of these complexities, AGR, by integrating both inflammatory and nutritional dimensions, may serve as a more stable and informative systemic marker for anticipating antitumor responses to immune checkpoint inhibition.

Moreover, emerging evidence has highlighted that systemic inflammatory and nutritional markers, including the AGR, are closely intertwined with the tumour immune microenvironment (TIME) [36]. A lower AGR, reflecting elevated globulin and inflammatory mediator levels, may correspond to an immunosuppressive TIME characterized by increased infiltration of tumour-associated macrophages, myeloid-derived suppressor cells, and regulatory T cells—cell populations known to attenuate cytotoxic T-cell activity and diminish the efficacy of ICIs. Conversely, a higher AGR may indicate a more balanced immune milieu with reduced pro-inflammatory cytokine signalling and enhanced antigen presentation, thereby facilitating T-cell–mediated antitumor responses. Recent reviews have emphasized that modulation of both innate and adaptive immune cells within the TIME is critical for overcoming resistance to ICIs and optimizing immunotherapeutic outcomes [37]. Furthermore, advances in immunotherapy, including tumour vaccines and combination strategies targeting cytokine networks and antigen-presenting pathways, further underscore the pivotal role of systemic immune–metabolic status—of which AGR is a surrogate—in shaping responsiveness to checkpoint blockade [38]. These findings collectively support the mechanistic plausibility that AGR not only reflects general inflammation and nutritional state but also mirrors functional dynamics within the TIME, thereby influencing therapeutic efficacy.

Collectively, our findings position the AGR as a clinically meaningful and biologically plausible biomarker for predicting therapeutic outcomes in cancer patients receiving immune checkpoint inhibitors. By capturing the interplay between systemic inflammation and nutritional status—two critical determinants of host–tumour dynamics—AGR offers a composite measure that is not only easily accessible through standard laboratory testing but also mechanistically aligned with the immunomodulatory landscape shaped by ICIs. Elevated AGR appears to reflect a more favourable immunometabolic milieu, characterized by attenuated pro-inflammatory signalling and preserved physiological resilience, thereby supporting more durable responses to PD-1-targeted therapies. While intrinsic variability in albumin or globulin levels may arise from non-malignant conditions, the integrated nature of AGR enhances its robustness and clinical interpretability. Taken together, these insights underscore the translational potential of AGR as a stratification tool for guiding immunotherapeutic decision-making and optimizing personalized treatment paradigms in oncology.

Although this meta-analysis yields clinically meaningful insights, certain intrinsic limitations must be acknowledged. Foremost among them is the reliance solely on retrospective observational datasets, which may compromise the robustness and generalizability of the synthesized hazard estimates. Moreover, it should be noted that the majority of the included studies (six out of seven) were conducted in Asian populations, with only one study originating from Turkey. This geographic concentration may introduce regional or ethnic bias, potentially limiting the extrapolation of our findings to non-Asian populations.

In addition, the patient populations analysed in the included studies were predominantly treated with immune checkpoint inhibitors as second-line or later-line therapies, which may restrict the applicability of our findings to patients receiving ICIs in earlier treatment settings or in countries where first-line immunotherapy is accessible. Furthermore, additional adjustments for tumour pathological characteristics—such as PD-L1 expression levels and tumour mutational burden—were not feasible due to the lack of such data in most included studies. Heterogeneity arising from the inclusion of different cancer types and varying ICI regimens may also have influenced survival outcomes.

Finally, inconsistencies in the operational definition and cutoff determination of the AGR across studies introduce considerable methodological variability. To address these shortcomings, future investigations should be designed as prospective, multi-institutional trials employing harmonized AGR stratification criteria and incorporating comprehensive clinicopathological and molecular data. Such efforts are essential to substantiate the prognostic relevance of AGR and to facilitate its integration into routine oncologic risk assessment frameworks.

## 5. Conclusion

Collectively, our findings underscore the prognostic utility of the AGR as a minimally invasive, cost-effective, and readily accessible biomarker in the context of ICI therapy. Through comprehensive meta-analytical synthesis and validation in an independent RCC cohort, elevated pretreatment AGR levels were consistently associated with superior overall survival, progression-free survival, and disease control rate. These results suggest that AGR may reflect a favourable immuno-nutritional profile capable of modulating host–tumour immune dynamics, thereby influencing therapeutic responsiveness. Given its clinical feasibility and reproducibility, AGR holds promise as a valuable adjunct to existing stratification tools for optimizing patient selection and tailoring immunotherapeutic strategies. Prospective, multicentre studies are warranted to confirm these observations and further delineate the mechanistic underpinnings of AGR within the immuno-oncologic landscape.

## Supplementary Material

Supplemental Material

Figure S1.tif

Supplementary table 1.docx

PRISMA 2020 Checklist.docx

## Data Availability

The data that support the findings of this study are available from the corresponding author upon reasonable request.
