# Supplementary material for: Albumin–globulin ratio is a predictive biomarker of antitumour effect of immune checkpoint inhibitors in cancer patients
Source: Ann Med. 2025 Nov 24;57(1):2591219. doi: 10.1080/07853890.2025.2591219 (PMC12646090; doi:10.1080/07853890.2025.2591219)
Supplement: Supplemental Material [file IANN_A_2591219_SM1345.docx]

Supplementary material 1. PUBMED database search strategy

((Camrelizumab) OR (Sintilimab) OR (Tislelizumab) OR (Toripalimab) OR (Envafolimab) OR (Immune Checkpoint Inhibitors) OR (Checkpoint Inhibitors, Immune) OR (Immune Checkpoint Inhibitor) OR (Checkpoint Inhibitor, Immune) OR (Immune Checkpoint Blockers) OR (Checkpoint Blockers, Immune) OR (Immune Checkpoint Blockade) OR (Checkpoint Blockade, Immune) OR (Immune Checkpoint Inhibition) OR (Checkpoint Inhibition, Immune) OR (PD-L1 Inhibitors) OR (PD L1 Inhibitors) OR (PD-L1 Inhibitor) OR (PD L1 Inhibitor) OR (Programmed Death-Ligand 1 Inhibitors) OR (Programmed Death Ligand 1 Inhibitors) OR (PD-1-PD-L1 Blockade) OR (Blockade, PD-1-PD-L1) OR (PD 1 PD L1 Blockade) OR (CTLA-4 Inhibitors) OR (CTLA 4 Inhibitors) OR (CTLA-4 Inhibitor) OR (CTLA 4 Inhibitor) OR (Cytotoxic T-Lymphocyte-Associated Protein 4 Inhibitors) OR (Cytotoxic T Lymphocyte Associated Protein 4 Inhibitors) OR (Cytotoxic T-Lymphocyte-Associated Protein 4 Inhibitor) OR (Cytotoxic T Lymphocyte Associated Protein 4 Inhibitor) OR (PD-1 Inhibitors) OR (PD-1 Inhibitor) OR (PD 1 Inhibitors) OR (Inhibitor, PD-1) OR (PD 1 Inhibitor) OR (Programmed Cell Death Protein 1 Inhibitor) OR (Programmed Cell Death Protein 1 Inhibitors) OR (Pembrolizumab) OR (Nivolumab) OR (Atezolizumab) OR (Ipilimumab) OR (Avelumab) OR (Tremelimumab) OR (Durvalumab) OR (Cemiplimab) OR (Immune Checkpoint Inhibitors[MeSH Terms])) AND ((((“albumin-to-globulin ratio”) OR (“albumin-globulin ratio”)) OR (“albumin to globulin ratio”)) OR (“albumin/globulin ratio”) OR (“AGR”))

Figure S1. (A) Subgroup analysis based on the Cox model revealed the relationship between the baseline albumin-to-globulin ratio and overall survival of cancer patients treated with immune checkpoint inhibitors. (B) Subgroup analysis based on the Cox model revealed the relationship between the baseline albumin-to-globulin ratio and progression-free survival of cancer patients treated with immune checkpoint inhibitors. HR, hazard ratio; CI, confidence interval.
