# Supplementary figures and images for: Albumin–globulin ratio is a predictive biomarker of antitumour effect of immune checkpoint inhibitors in cancer patients
Source: Ann Med. 2025 Nov 24;57(1):2591219. doi: 10.1080/07853890.2025.2591219 (PMC12646090; doi:10.1080/07853890.2025.2591219)

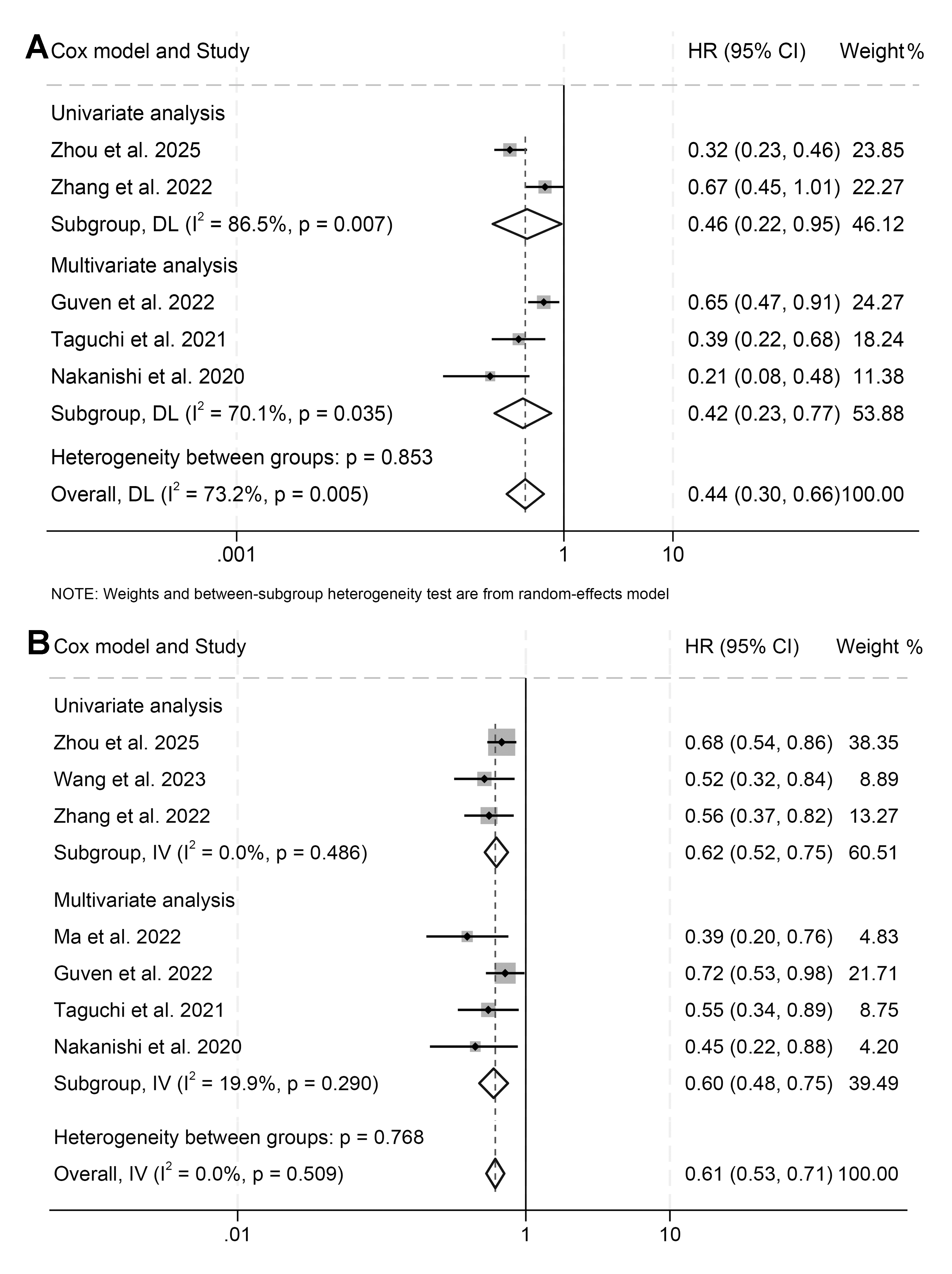

Supplement: Figure S1.tif [file IANN_A_2591219_SM2643.tif]
