# Supplementary material for: Albumin–globulin ratio is a predictive biomarker of antitumour effect of immune checkpoint inhibitors in cancer patients
Source: Ann Med. 2025 Nov 24;57(1):2591219. doi: 10.1080/07853890.2025.2591219 (PMC12646090; doi:10.1080/07853890.2025.2591219)
Supplement: Supplementary table 1.docx [file IANN_A_2591219_SM2642.docx]

| Supplementary table 1. Patient characteristics | |
| --- | --- |
|  | Overall (n=74) |
| Age | 56.4 (34.7-79.6) |
| Males | 50 (67.57%) |
| ECOG PS |  |
| 0 | 40 (54.06%) |
| 1 | 25 (33.78%) |
| 2 | 9 (12.16%) |
| IMDC risk group |  |
| 0 | 38 (51.35%) |
| 1 | 27 (36.49%) |
| ≥ 2 | 9 (12.16%) |
| Treatment line |  |
| First-line | 30 (40.54%) |
| Later-line | 44 (59.46%) |
| Data shown are means with range or numbers with percentage. ECOG PS, Eastern Cooperative Oncology Group performance status; IMDC, International Metastatic RCC Database Consortium | |
